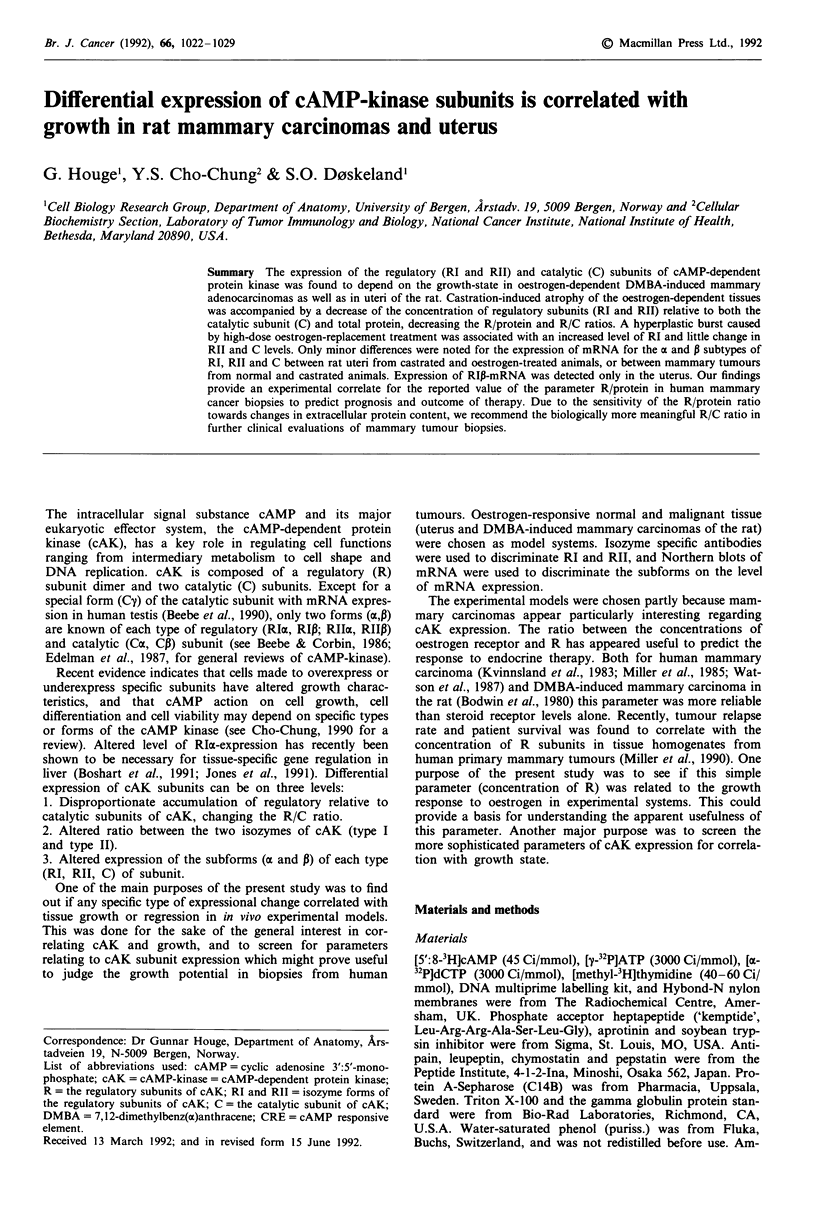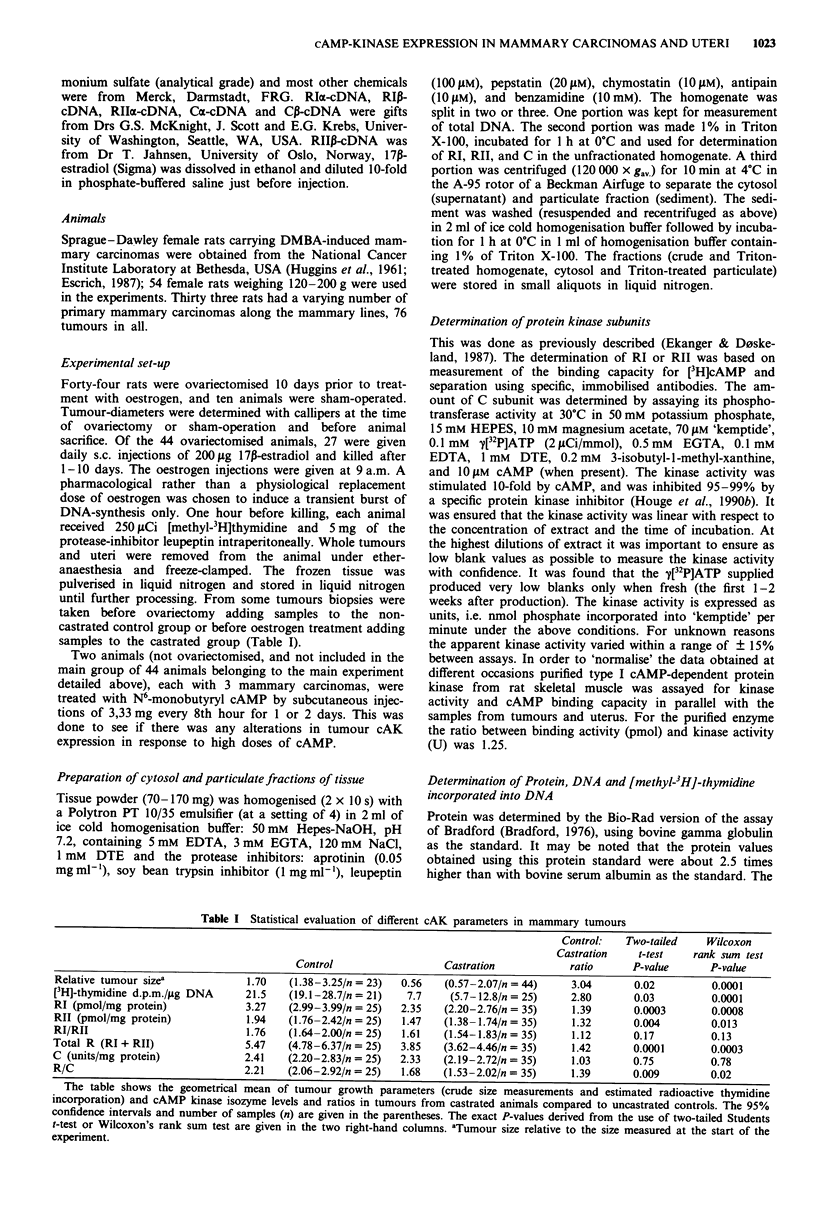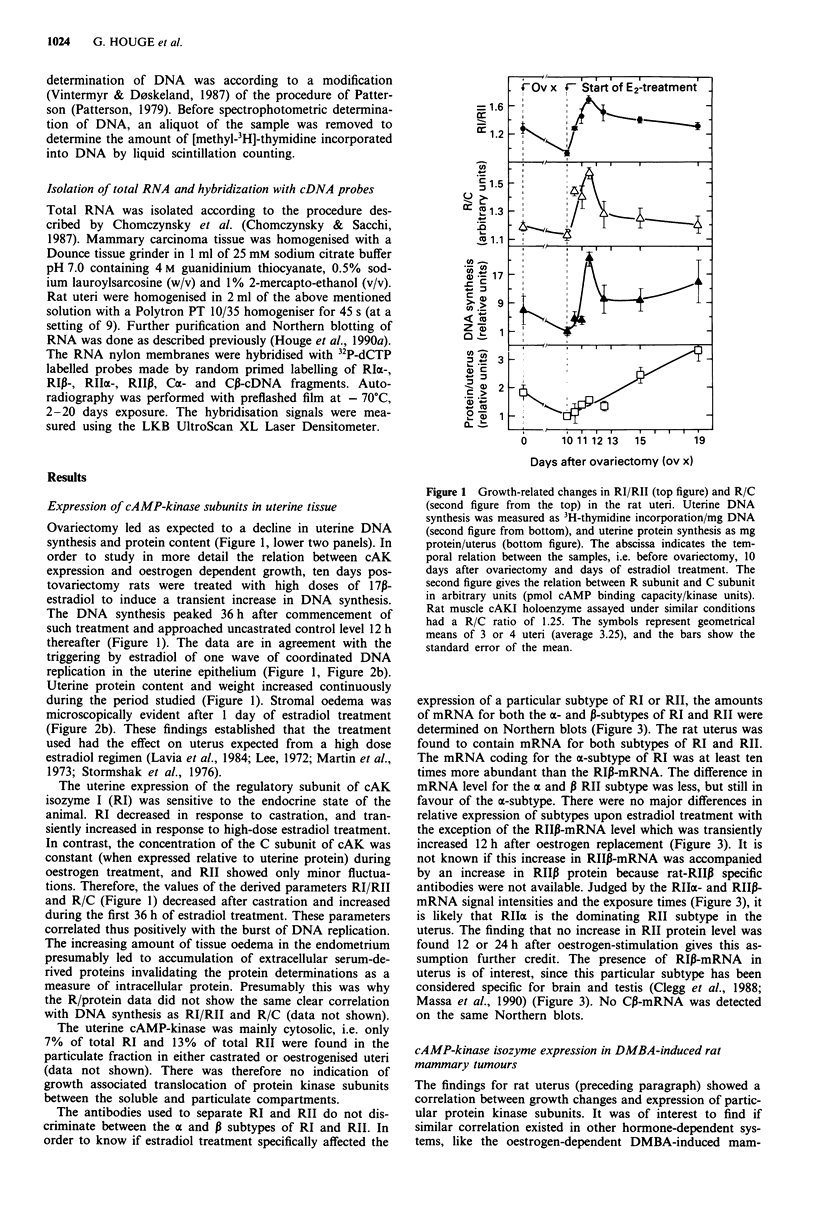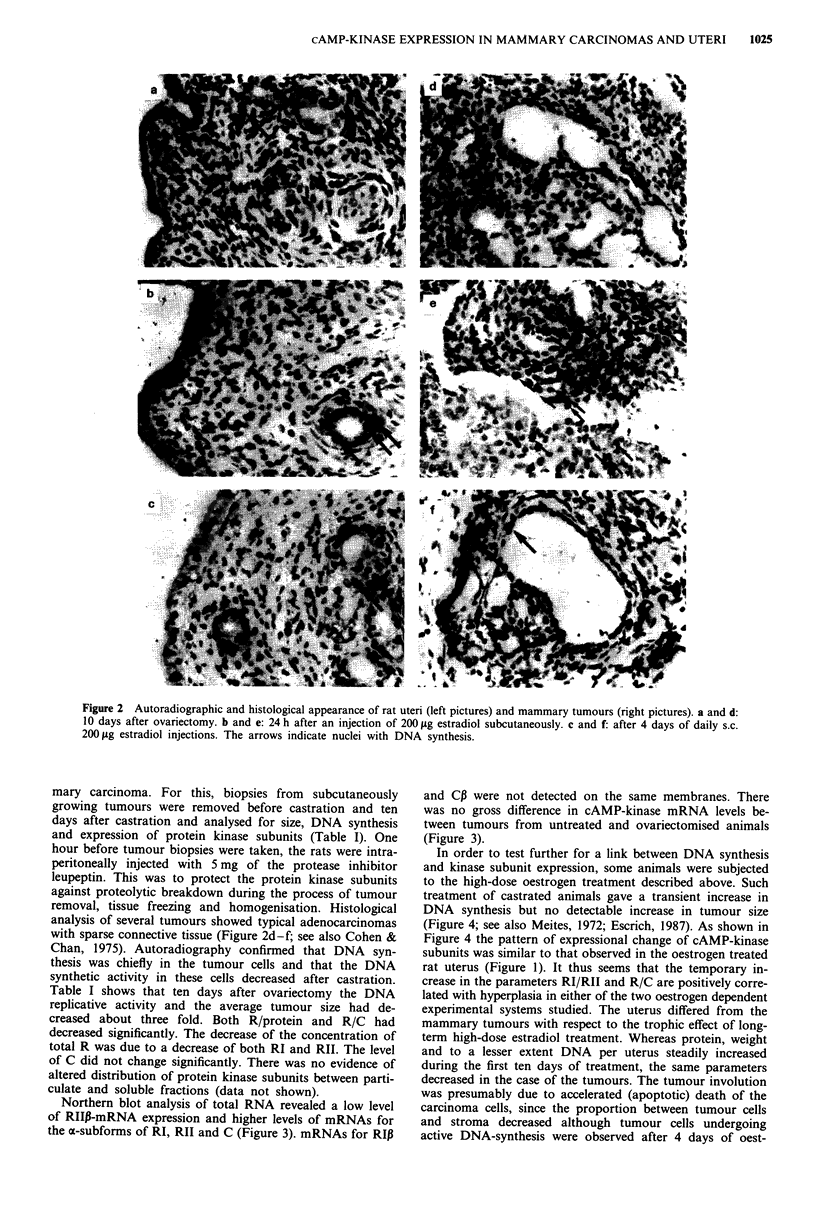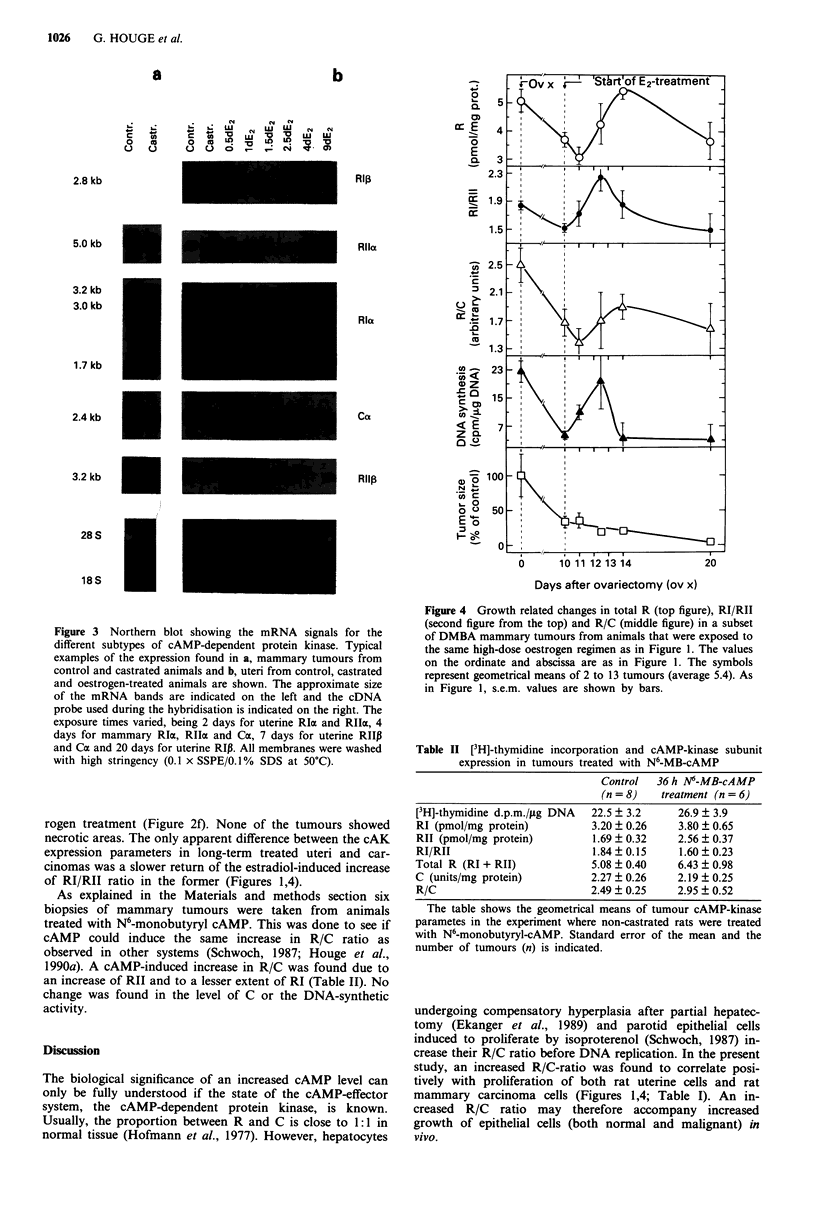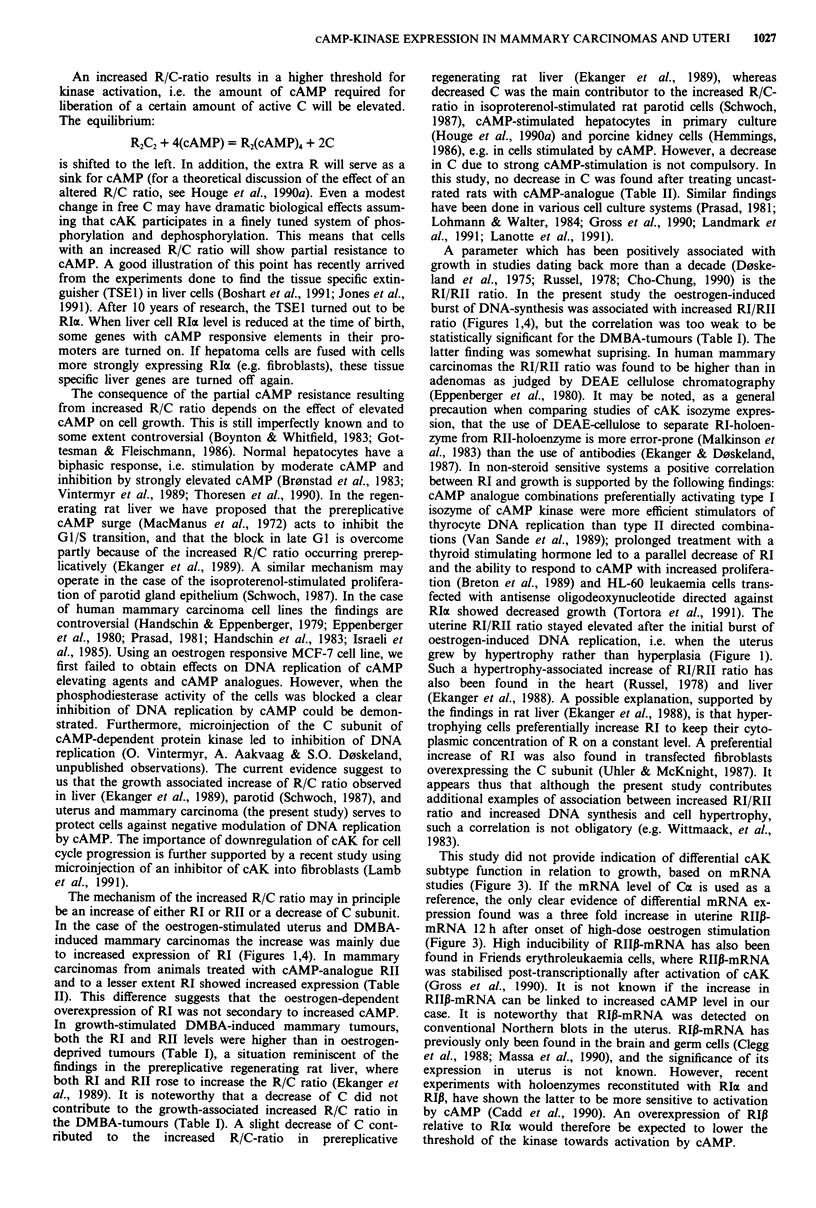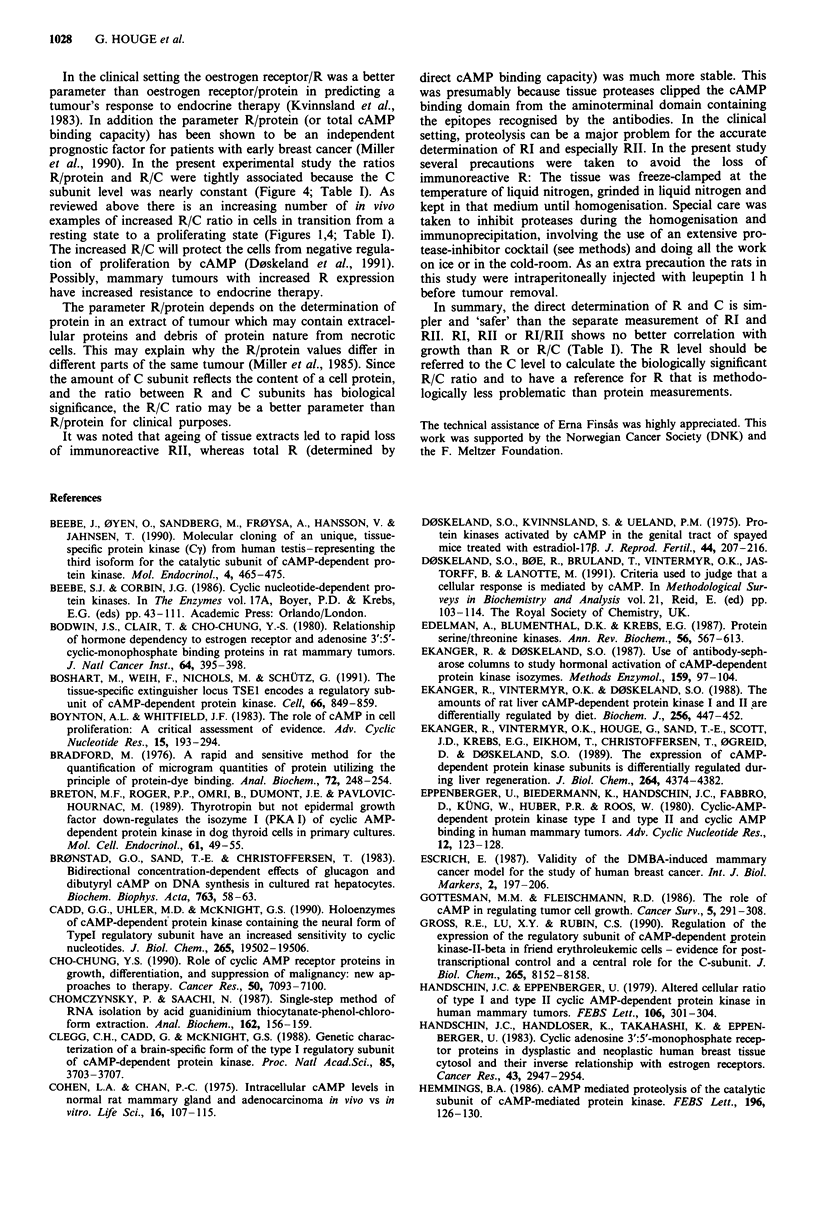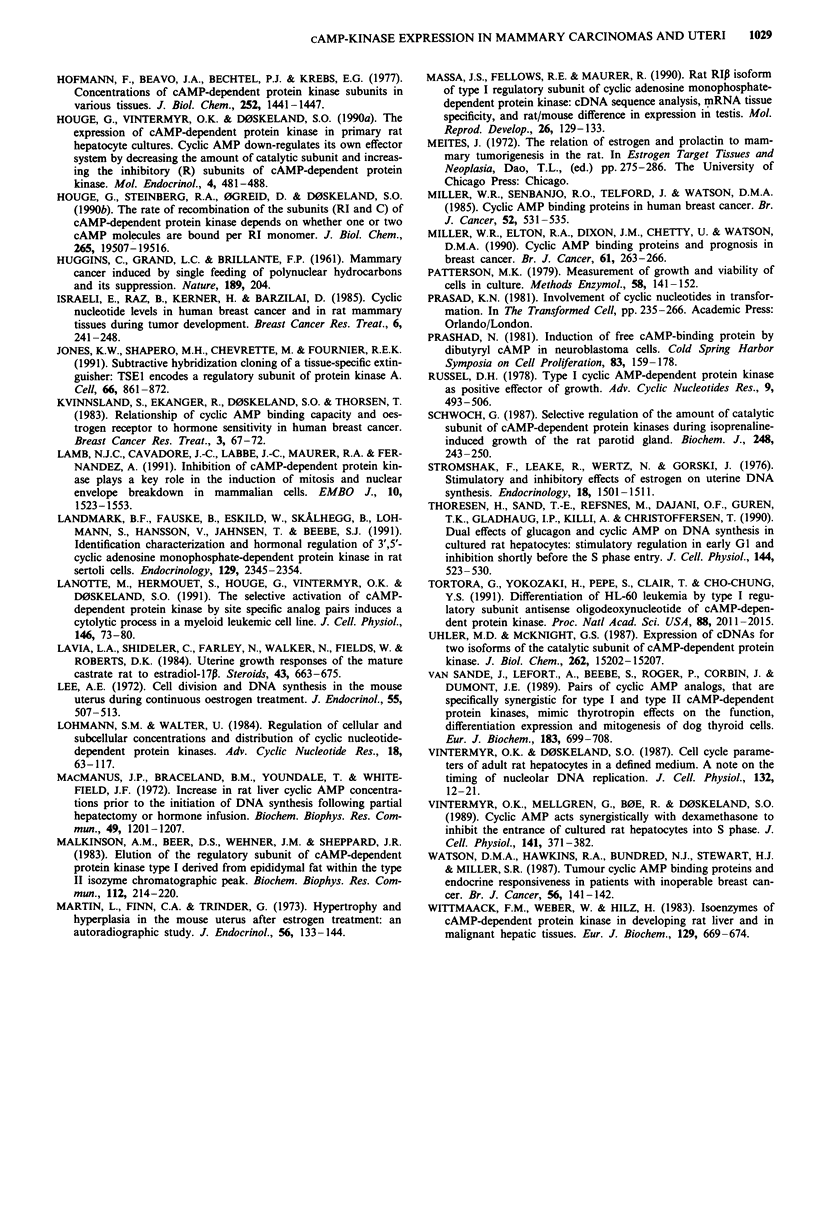# Differential expression of cAMP-kinase subunits is correlated with growth in rat mammary carcinomas and uterus.

**DOI:** 10.1038/bjc.1992.404

**Published:** 1992-12

**Authors:** G. Houge, Y. S. Cho-Chung, S. O. Døskeland

**Affiliations:** Department of Anatomy, University of Bergen, Arstadv., Norway.

## Abstract

**Images:**


*Br. J. Cancer* (1992), **66**, 1022–1029 © Macmillan Press Ltd., 1992

### Differential expression of cAMP-kinase subunits is correlated with growth in rat mammary carcinomas and uterus

G. Houge[1], Y.S. Cho-Chung[2] & S.O. Døskeland[1]

[1]*Cell Biology Research Group, Department of Anatomy, University of Bergen, Årstadv. 19, 5009 Bergen, Norway and* [2]*Cellular Biochemistry Section, Laboratory of Tumor Immunology and Biology, National Cancer Institute, National Institute of Health, Bethesda, Maryland 20890, USA.*

**Summary** The expression of the regulatory (RI and RII) and catalytic (C) subunits of cAMP-dependent protein kinase was found to depend on the growth-state in oestrogen-dependent DMBA-induced mammary adenocarcinomas as well as in uteri of the rat. Castration-induced atrophy of the oestrogen-dependent tissues was accompanied by a decrease of the concentration of regulatory subunits (RI and RII) relative to both the catalytic subunit (C) and total protein, decreasing the R/protein and R/C ratios. A hyperplastic burst caused by high-dose oestrogen-replacement treatment was associated with an increased level of RI and little change in RII and C levels. Only minor differences were noted for the expression of mRNA for the α and β subtypes of RI, RII and C between rat uteri from castrated and oestrogen-treated animals, or between mammary tumours from normal and castrated animals. Expression of RIβ-mRNA was detected only in the uterus. Our findings provide an experimental correlate for the reported value of the parameter R/protein in human mammary cancer biopsies to predict prognosis and outcome of therapy. Due to the sensitivity of the R/protein ratio towards changes in extracellular protein content, we recommend the biologically more meaningful R/C ratio in further clinical evaluations of mammary tumour biopsies.

The intracellular signal substance cAMP and its major eukaryotic effector system, the cAMP-dependent protein kinase (cAK), has a key role in regulating cell functions ranging from intermediary metabolism to cell shape and DNA replication. cAK is composed of a regulatory (R) subunit dimer and two catalytic (C) subunits. Except for a special form (Cγ) of the catalytic subunit with mRNA expression in human testis (Beebe *et al.*, 1990), only two forms (α,β) are known of each type of regulatory (RIα, RIβ; RIIα, RIIβ) and catalytic (Cα, Cβ) subunit (see Beebe & Corbin, 1986; Edelman *et al.*, 1987, for general reviews of cAMP-kinase).

Recent evidence indicates that cells made to overexpress or underexpress specific subunits have altered growth characteristics, and that cAMP action on cell growth, cell differentiation and cell viability may depend on specific types or forms of the cAMP kinase (see Cho-Chung, 1990 for a review). Altered level of RIα-expression has recently been shown to be necessary for tissue-specific gene regulation in liver (Boshart *et al.*, 1991; Jones *et al.*, 1991). Differential expression of cAK subunits can be on three levels:

1. Disproportionate accumulation of regulatory relative to catalytic subunits of cAK, changing the R/C ratio.
2. Altered ratio between the two isozymes of cAK (type I and type II).
3. Altered expression of the subforms (α and β) of each type (RI, RII, C) of subunit.

One of the main purposes of the present study was to find out if any specific type of expressional change correlated with tissue growth or regression in *in vivo* experimental models. This was done for the sake of the general interest in correlating cAK and growth, and to screen for parameters relating to cAK subunit expression which might prove useful to judge the growth potential in biopsies from human tumours. Oestrogen-responsive normal and malignant tissue (uterus and DMBA-induced mammary carcinomas of the rat) were chosen as model systems. Isozyme specific antibodies were used to discriminate RI and RII, and Northern blots of mRNA were used to discriminate the subforms on the level of mRNA expression.

The experimental models were chosen partly because mammary carcinomas appear particularly interesting regarding cAK expression. The ratio between the concentrations of oestrogen receptor and R has appeared useful to predict the response to endocrine therapy. Both for human mammary carcinoma (Kvinnsland *et al.*, 1983; Miller *et al.*, 1985; Watson *et al.*, 1987) and DMBA-induced mammary carcinoma in the rat (Bodwin *et al.*, 1980) this parameter was more reliable than steroid receptor levels alone. Recently, tumour relapse rate and patient survival was found to correlate with the concentration of R subunits in tissue homogenates from human primary mammary tumours (Miller *et al.*, 1990). One purpose of the present study was to see if this simple parameter (concentration of R) was related to the growth response to oestrogen in experimental systems. This could provide a basis for understanding the apparent usefulness of this parameter. Another major purpose was to screen the more sophisticated parameters of cAK expression for correlation with growth state.

### Materials and methods

### *Materials*

[5′:8-$^{3}$H]cAMP (45 Ci/mmol), [γ-$^{32}$P]ATP (3000 Ci/mmol), [α-$^{32}$P]dCTP (3000 Ci/mmol), [methyl-$^{3}$H]thymidine (40–60 Ci/mmol), DNA multiprime labelling kit, and Hybond-N nylon membranes were from The Radiochemical Centre, Amersham, UK. Phosphate acceptor heptapeptide ('kemptide', Leu-Arg-Arg-Ala-Ser-Leu-Gly), aprotinin and soybean trypsin inhibitor were from Sigma, St. Louis, MO, USA. Antipain, leupeptin, chymostatin and pepstatin were from the Peptide Institute, 4-1-2-Ina, Minoshi, Osaka 562, Japan. Protein A-Sepharose (C14B) was from Pharmacia, Uppsala, Sweden. Triton X-100 and the gamma globulin protein standard were from Bio-Rad Laboratories, Richmond, CA, U.S.A. Water-saturated phenol (puriss.) was from Fluka, Buchs, Switzerland, and was not redistilled before use. Am-

Correspondence: Dr Gunnar Houge, Department of Anatomy, Årstadveien 19, N-5009 Bergen, Norway.

List of abbreviations used: cAMP = cyclic adenosine 3′:5′-monophosphate; cAK = cAMP-kinase = cAMP-dependent protein kinase; R = the regulatory subunits of cAK; RI and RII = isozyme forms of the regulatory subunits of cAK; C = the catalytic subunit of cAK; DMBA = 7,12-dimethylbenz(α)anthracene; CRE = cAMP responsive element.

Received 13 March 1992; and in revised form 15 June 1992.

monium sulfate (analytical grade) and most other chemicals were from Merck, Darmstadt, FRG. RIα-cDNA, RIβ-cDNA, RIIα-cDNA, Cα-cDNA and Cβ-cDNA were gifts from Drs G.S. McKnight, J. Scott and E.G. Krebs, University of Washington, Seattle, WA, USA. RIIβ-cDNA was from Dr T. Jahnsen, University of Oslo, Norway, 17β-estradiol (Sigma) was dissolved in ethanol and diluted 10-fold in phosphate-buffered saline just before injection.

*Animals*

Sprague-Dawley female rats carrying DMBA-induced mammary carcinomas were obtained from the National Cancer Institute Laboratory at Bethesda, USA (Huggins *et al.*, 1961; Escrich, 1987); 54 female rats weighing 120-200 g were used in the experiments. Thirty three rats had a varying number of primary mammary carcinomas along the mammary lines, 76 tumours in all.

*Experimental set-up*

Forty-four rats were ovariectomised 10 days prior to treatment with oestrogen, and ten animals were sham-operated. Tumour-diameters were determined with callipers at the time of ovariectomy or sham-operation and before animal sacrifice. Of the 44 ovariectomised animals, 27 were given daily s.c. injections of 200 μg 17β-estradiol and killed after 1-10 days. The oestrogen injections were given at 9 a.m. A pharmacological rather than a physiological replacement dose of oestrogen was chosen to induce a transient burst of DNA-synthesis only. One hour before killing, each animal received 250 μCi [methyl-$^3$H]thymidine and 5 mg of the protease-inhibitor leupeptin intraperitoneally. Whole tumours and uteri were removed from the animal under ether-anaesthesia and freeze-clamped. The frozen tissue was pulverised in liquid nitrogen and stored in liquid nitrogen until further processing. From some tumours biopsies were taken before ovariectomy adding samples to the non-castrated control group or before oestrogen treatment adding samples to the castrated group (Table I).

Two animals (not ovariectomised, and not included in the main group of 44 animals belonging to the main experiment detailed above), each with 3 mammary carcinomas, were treated with N$^6$-monobutyryl cAMP by subcutaneous injections of 3,33 mg every 8th hour for 1 or 2 days. This was done to see if there was any alterations in tumour cAK expression in response to high doses of cAMP.

*Preparation of cytosol and particulate fractions of tissue*

Tissue powder (70-170 mg) was homogenised (2 × 10 s) with a Polytron PT 10/35 emulsifier (at a setting of 4) in 2 ml of ice cold homogenisation buffer: 50 mM Hepes-NaOH, pH 7.2, containing 5 mM EDTA, 3 mM EGTA, 120 mM NaCl, 1 mM DTE and the protease inhibitors: aprotinin (0.05 mg ml$^{-1}$), soy bean trypsin inhibitor (1 mg ml$^{-1}$), leupeptin (100 μM), pepstatin (20 μM), chymostatin (10 μM), antipain (10 μM), and benzamidine (10 mM). The homogenate was split in two or three. One portion was kept for measurement of total DNA. The second portion was made 1% in Triton X-100, incubated for 1 h at 0°C and used for determination of RI, RII, and C in the unfractionated homogenate. A third portion was centrifuged (120 000 × $g_{av.}$) for 10 min at 4°C in the A-95 rotor of a Beckman Airfuge to separate the cytosol (supernatant) and particulate fraction (sediment). The sediment was washed (resuspended and recentrifuged as above) in 2 ml of ice cold homogenisation buffer followed by incubation for 1 h at 0°C in 1 ml of homogenisation buffer containing 1% of Triton X-100. The fractions (crude and Triton-treated homogenate, cytosol and Triton-treated particulate) were stored in small aliquots in liquid nitrogen.

*Determination of protein kinase subunits*

This was done as previously described (Ekanger & Døskeland, 1987). The determination of RI or RII was based on measurement of the binding capacity for [$^3$H]cAMP and separation using specific, immobilised antibodies. The amount of C subunit was determined by assaying its phosphotransferase activity at 30°C in 50 mM potassium phosphate, 15 mM HEPES, 10 mM magnesium acetate, 70 μM 'kemptide', 0.1 mM γ[$^{32}$P]ATP (2 μCi/mmol), 0.5 mM EGTA, 0.1 mM EDTA, 1 mM DTE, 0.2 mM 3-isobutyl-1-methyl-xanthine, and 10 μM cAMP (when present). The kinase activity was stimulated 10-fold by cAMP, and was inhibited 95-99% by a specific protein kinase inhibitor (Houge *et al.*, 1990*b*). It was ensured that the kinase activity was linear with respect to the concentration of extract and the time of incubation. At the highest dilutions of extract it was important to ensure as low blank values as possible to measure the kinase activity with confidence. It was found that the γ[$^{32}$P]ATP supplied produced very low blanks only when fresh (the first 1-2 weeks after production). The kinase activity is expressed as units, i.e. nmol phosphate incorporated into 'kemptide' per minute under the above conditions. For unknown reasons the apparent kinase activity varied within a range of ± 15% between assays. In order to 'normalise' the data obtained at different occasions purified type I cAMP-dependent protein kinase from rat skeletal muscle was assayed for kinase activity and cAMP binding capacity in parallel with the samples from tumours and uterus. For the purified enzyme the ratio between binding activity (pmol) and kinase activity (U) was 1.25.

*Determination of Protein, DNA and [methyl-$^3$H]-thymidine incorporated into DNA*

Protein was determined by the Bio-Rad version of the assay of Bradford (Bradford, 1976), using bovine gamma globulin as the standard. It may be noted that the protein values obtained using this protein standard were about 2.5 times higher than with bovine serum albumin as the standard. The

**Table I** Statistical evaluation of different cAK parameters in mammary tumours

| | Control | | Castration | | *Control: Castration ratio* | *Two-tailed t-test P-value* | *Wilcoxon rank sum test P-value* |
|---|---|---|---|---|---|---|---|
| Relative tumour size[a] | 1.70 | (1.38-3.25/*n* = 23) | 0.56 | (0.57-2.07/*n* = 44) | 3.04 | 0.02 | 0.0001 |
| [$^3$H]-thymidine d.p.m./μg DNA | 21.5 | (19.1-28.7/*n* = 21) | 7.7 | (5.7-12.8/*n* = 25) | 2.80 | 0.03 | 0.0001 |
| RI (pmol/mg protein) | 3.27 | (2.99-3.99/*n* = 25) | 2.35 | (2.20-2.76/*n* = 35) | 1.39 | 0.0003 | 0.0008 |
| RII (pmol/mg protein) | 1.94 | (1.76-2.42/*n* = 25) | 1.47 | (1.38-1.74/*n* = 35) | 1.32 | 0.004 | 0.013 |
| RI/RII | 1.76 | (1.64-2.00/*n* = 25) | 1.61 | (1.54-1.83/*n* = 35) | 1.12 | 0.17 | 0.13 |
| Total R (RI + RII) | 5.47 | (4.78-6.37/*n* = 25) | 3.85 | (3.62-4.46/*n* = 35) | 1.42 | 0.0001 | 0.0003 |
| C (units/mg protein) | 2.41 | (2.20-2.83/*n* = 25) | 2.33 | (2.19-2.72/*n* = 35) | 1.03 | 0.75 | 0.78 |
| R/C | 2.21 | (2.06-2.92/*n* = 25) | 1.68 | (1.53-2.02/*n* = 35) | 1.39 | 0.009 | 0.02 |

The table shows the geometrical mean of tumour growth parameters (crude size measurements and estimated radioactive thymidine incorporation) and cAMP kinase isozyme levels and ratios in tumours from castrated animals compared to uncastrated controls. The 95% confidence intervals and number of samples (*n*) are given in the parentheses. The exact *P*-values derived from the use of two-tailed Students *t*-test or Wilcoxon's rank sum test are given in the two right-hand columns. [a]Tumour size relative to the size measured at the start of the experiment.

determination of DNA was according to a modification (Vintermyr & Døskeland, 1987) of the procedure of Patterson (Patterson, 1979). Before spectrophotometric determination of DNA, an aliquot of the sample was removed to determine the amount of [methyl-$^3$H]-thymidine incorporated into DNA by liquid scintillation counting.

*Isolation of total RNA and hybridization with cDNA probes*

Total RNA was isolated according to the procedure described by Chomczynsky et al. (Chomczynsky & Sacchi, 1987). Mammary carcinoma tissue was homogenised with a Dounce tissue grinder in 1 ml of 25 mM sodium citrate buffer pH 7.0 containing 4 M guanidinium thiocyanate, 0.5% sodium lauroylsarcosine (w/v) and 1% 2-mercapto-ethanol (v/v). Rat uteri were homogenised in 2 ml of the above mentioned solution with a Polytron PT 10/35 homogeniser for 45 s (at a setting of 9). Further purification and Northern blotting of RNA was done as described previously (Houge et al., 1990a). The RNA nylon membranes were hybridised with $^{32}$P-dCTP labelled probes made by random primed labelling of RIα-, RIβ-, RIIα-, RIIβ, Cα- and Cβ-cDNA fragments. Autoradiography was performed with preflashed film at − 70°C, 2–20 days exposure. The hybridisation signals were measured using the LKB UltroScan XL Laser Densitometer.

**Figure 1** Growth-related changes in RI/RII (top figure) and R/C (second figure from the top) in the rat uteri. Uterine DNA synthesis was measured as $^3$H-thymidine incorporation/mg DNA (second figure from bottom), and uterine protein synthesis as mg protein/uterus (bottom figure). The abscissa indicates the temporal relation between the samples, i.e. before ovariectomy, 10 days after ovariectomy and days of estradiol treatment. The second figure gives the relation between R subunit and C subunit in arbitrary units (pmol cAMP binding capacity/kinase units). Rat muscle cAKI holoenzyme assayed under similar conditions had a R/C ratio of 1.25. The symbols represent geometrical means of 3 or 4 uteri (average 3.25), and the bars show the standard error of the mean.

### Results

*Expression of cAMP-kinase subunits in uterine tissue*

Ovariectomy led as expected to a decline in uterine DNA synthesis and protein content (Figure 1, lower two panels). In order to study in more detail the relation between cAK expression and oestrogen dependent growth, ten days postovariectomy rats were treated with high doses of 17β-estradiol to induce a transient increase in DNA synthesis. The DNA synthesis peaked 36 h after commencement of such treatment and approached uncastrated control level 12 h thereafter (Figure 1). The data are in agreement with the triggering by estradiol of one wave of coordinated DNA replication in the uterine epithelium (Figure 1, Figure 2b). Uterine protein content and weight increased continuously during the period studied (Figure 1). Stromal oedema was microscopically evident after 1 day of estradiol treatment (Figure 2b). These findings established that the treatment used had the effect on uterus expected from a high dose estradiol regimen (Lavia et al., 1984; Lee, 1972; Martin et al., 1973; Stormshak et al., 1976).

The uterine expression of the regulatory subunit of cAK isozyme I (RI) was sensitive to the endocrine state of the animal. RI decreased in response to castration, and transiently increased in response to high-dose estradiol treatment. In contrast, the concentration of the C subunit of cAK was constant (when expressed relative to uterine protein) during oestrogen treatment, and RII showed only minor fluctuations. Therefore, the values of the derived parameters RI/RII and R/C (Figure 1) decreased after castration and increased during the first 36 h of estradiol treatment. These parameters correlated thus positively with the burst of DNA replication. The increasing amount of tissue oedema in the endometrium presumably led to accumulation of extracellular serum-derived proteins invalidating the protein determinations as a measure of intracellular protein. Presumably this was why the R/protein data did not show the same clear correlation with DNA synthesis as RI/RII and R/C (data not shown).

The uterine cAMP-kinase was mainly cytosolic, i.e. only 7% of total RI and 13% of total RII were found in the particulate fraction in either castrated or oestrogenised uteri (data not shown). There was therefore no indication of growth associated translocation of protein kinase subunits between the soluble and particulate compartments.

The antibodies used to separate RI and RII do not discriminate between the α and β subtypes of RI and RII. In order to know if estradiol treatment specifically affected the expression of a particular subtype of RI or RII, the amounts of mRNA for both the α- and β-subtypes of RI and RII were determined on Northern blots (Figure 3). The rat uterus was found to contain mRNA for both subtypes of RI and RII. The mRNA coding for the α-subtype of RI was at least ten times more abundant than the RIβ-mRNA. The difference in mRNA level for the α and β RII subtype was less, but still in favour of the α-subtype. There were no major differences in relative expression of subtypes upon estradiol treatment with the exception of the RIIβ-mRNA level which was transiently increased 12 h after oestrogen replacement (Figure 3). It is not known if this increase in RIIβ-mRNA was accompanied by an increase in RIIβ protein because rat-RIIβ specific antibodies were not available. Judged by the RIIα- and RIIβ-mRNA signal intensities and the exposure times (Figure 3), it is likely that RIIα is the dominating RII subtype in the uterus. The finding that no increase in RII protein level was found 12 or 24 h after oestrogen-stimulation gives this assumption further credit. The presence of RIβ-mRNA in uterus is of interest, since this particular subtype has been considered specific for brain and testis (Clegg et al., 1988; Massa et al., 1990) (Figure 3). No Cβ-mRNA was detected on the same Northern blots.

*cAMP-kinase isozyme expression in DMBA-induced rat mammary tumours*

The findings for rat uterus (preceding paragraph) showed a correlation between growth changes and expression of particular protein kinase subunits. It was of interest to find if similar correlation existed in other hormone-dependent systems, like the oestrogen-dependent DMBA-induced mam-

**Figure 2** Autoradiographic and histological appearance of rat uteri (left pictures) and mammary tumours (right pictures). **a** and **d**: 10 days after ovariectomy. **b** and **e**: 24 h after an injection of 200 μg estradiol subcutaneously. **c** and **f**: after 4 days of daily s.c. 200 μg estradiol injections. The arrows indicate nuclei with DNA synthesis.

mary carcinoma. For this, biopsies from subcutaneously growing tumours were removed before castration and ten days after castration and analysed for size, DNA synthesis and expression of protein kinase subunits (Table I). One hour before tumour biopsies were taken, the rats were intraperitoneally injected with 5 mg of the protease inhibitor leupeptin. This was to protect the protein kinase subunits against proteolytic breakdown during the process of tumour removal, tissue freezing and homogenisation. Histological analysis of several tumours showed typical adenocarcinomas with sparse connective tissue (Figure 2d–f; see also Cohen & Chan, 1975). Autoradiography confirmed that DNA synthesis was chiefly in the tumour cells and that the DNA synthetic activity in these cells decreased after castration. Table I shows that ten days after ovariectomy the DNA replicative activity and the average tumour size had decreased about three fold. Both R/protein and R/C had decreased significantly. The decrease of the concentration of total R was due to a decrease of both RI and RII. The level of C did not change significantly. There was no evidence of altered distribution of protein kinase subunits between particulate and soluble fractions (data not shown).

Northern blot analysis of total RNA revealed a low level of RIIβ-mRNA expression and higher levels of mRNAs for the α-subforms of RI, RII and C (Figure 3). mRNAs for RIβ and Cβ were not detected on the same membranes. There was no gross difference in cAMP-kinase mRNA levels between tumours from untreated and ovariectomised animals (Figure 3).

In order to test further for a link between DNA synthesis and kinase subunit expression, some animals were subjected to the high-dose oestrogen treatment described above. Such treatment of castrated animals gave a transient increase in DNA synthesis but no detectable increase in tumour size (Figure 4; see also Meites, 1972; Escrich, 1987). As shown in Figure 4 the pattern of expressional change of cAMP-kinase subunits was similar to that observed in the oestrogen treated rat uterus (Figure 1). It thus seems that the temporary increase in the parameters RI/RII and R/C are positively correlated with hyperplasia in either of the two oestrogen dependent experimental systems studied. The uterus differed from the mammary tumours with respect to the trophic effect of long-term high-dose estradiol treatment. Whereas protein, weight and to a lesser extent DNA per uterus steadily increased during the first ten days of treatment, the same parameters decreased in the case of the tumours. The tumour involution was presumably due to accelerated (apoptotic) death of the carcinoma cells, since the proportion between tumour cells and stroma decreased although tumour cells undergoing active DNA-synthesis were observed after 4 days of oest-

Figure 3 Northern blot showing the mRNA signals for the different subtypes of cAMP-dependent protein kinase. Typical examples of the expression found in **a**, mammary tumours from control and castrated animals and **b**, uteri from control, castrated and oestrogen-treated animals are shown. The approximate size of the mRNA bands are indicated on the left and the cDNA probe used during the hybridisation is indicated on the right. The exposure times varied, being 2 days for uterine RIα and RIIα, 4 days for mammary RIα, RIIα and Cα, 7 days for uterine RIIβ and Cα and 20 days for uterine RIβ. All membranes were washed with high stringency (0.1 × SSPE/0.1% SDS at 50°C).

Figure 4 Growth related changes in total R (top figure), RI/RII (second figure from the top) and R/C (middle figure) in a subset of DMBA mammary tumours from animals that were exposed to the same high-dose oestrogen regimen as in Figure 1. The values on the ordinate and abscissa are as in Figure 1. The symbols represent geometrical means of 2 to 13 tumours (average 5.4). As in Figure 1, s.e.m. values are shown by bars.

rogen treatment (Figure 2f). None of the tumours showed necrotic areas. The only apparent difference between the cAK expression parameters in long-term treated uteri and carcinomas was a slower return of the estradiol-induced increase of RI/RII ratio in the former (Figures 1,4).

As explained in the Materials and methods section six biopsies of mammary tumours were taken from animals treated with $N^6$-monobutyryl cAMP. This was done to see if cAMP could induce the same increase in R/C ratio as observed in other systems (Schwoch, 1987; Houge *et al.*, 1990*a*). A cAMP-induced increase in R/C was found due to an increase of RII and to a lesser extent of RI (Table II). No change was found in the level of C or the DNA-synthetic activity.

**Table II** [$^3$H]-thymidine incorporation and cAMP-kinase subunit expression in tumours treated with $N^6$-MB-cAMP

| | *Control (n = 8)* | *36 h $N^6$-MB-cAMP treatment (n = 6)* |
|---|---|---|
| [$^3$H]-thymidine d.p.m./μg DNA | 22.5 ± 3.2 | 26.9 ± 3.9 |
| RI (pmol/mg protein) | 3.20 ± 0.26 | 3.80 ± 0.65 |
| RII (pmol/mg protein) | 1.69 ± 0.32 | 2.56 ± 0.37 |
| RI/RII | 1.84 ± 0.15 | 1.60 ± 0.23 |
| Total R (RI + RII) | 5.08 ± 0.40 | 6.43 ± 0.98 |
| C (units/mg protein) | 2.27 ± 0.26 | 2.19 ± 0.25 |
| R/C | 2.49 ± 0.25 | 2.95 ± 0.52 |

The table shows the geometrical means of tumour cAMP-kinase parametes in the experiment where non-castrated rats were treated with $N^6$-monobutyryl-cAMP. Standard error of the mean and the number of tumours (*n*) is indicated.

### Discussion

The biological significance of an increased cAMP level can only be fully understood if the state of the cAMP-effector system, the cAMP-dependent protein kinase, is known. Usually, the proportion between R and C is close to 1:1 in normal tissue (Hofmann *et al.*, 1977). However, hepatocytes undergoing compensatory hyperplasia after partial hepatectomy (Ekanger *et al.*, 1989) and parotid epithelial cells induced to proliferate by isoproterenol (Schwoch, 1987) increase their R/C ratio before DNA replication. In the present study, an increased R/C-ratio was found to correlate positively with proliferation of both rat uterine cells and rat mammary carcinoma cells (Figures 1,4; Table I). An increased R/C ratio may therefore accompany increased growth of epithelial cells (both normal and malignant) *in vivo*.

An increased R/C-ratio results in a higher threshold for kinase activation, i.e. the amount of cAMP required for liberation of a certain amount of active C will be elevated. The equilibrium:

$$R_2C_2 + 4(cAMP) = R_2(cAMP)_4 + 2C$$

is shifted to the left. In addition, the extra R will serve as a sink for cAMP (for a theoretical discussion of the effect of an altered R/C ratio, see Houge *et al.*, 1990*a*). Even a modest change in free C may have dramatic biological effects assuming that cAK participates in a finely tuned system of phosphorylation and dephosphorylation. This means that cells with an increased R/C ratio will show partial resistance to cAMP. A good illustration of this point has recently arrived from the experiments done to find the tissue specific extinguisher (TSE1) in liver cells (Boshart *et al.*, 1991; Jones *et al.*, 1991). After 10 years of research, the TSE1 turned out to be RIα. When liver cell RIα level is reduced at the time of birth, some genes with cAMP responsive elements in their promoters are turned on. If hepatoma cells are fused with cells more strongly expressing RIα (e.g. fibroblasts), these tissue specific liver genes are turned off again.

The consequence of the partial cAMP resistance resulting from increased R/C ratio depends on the effect of elevated cAMP on cell growth. This is still imperfectly known and to some extent controversial (Boynton & Whitfield, 1983; Gottesman & Fleischmann, 1986). Normal hepatocytes have a biphasic response, i.e. stimulation by moderate cAMP and inhibition by strongly elevated cAMP (Brønstad *et al.*, 1983; Vintermyr *et al.*, 1989; Thoresen *et al.*, 1990). In the regenerating rat liver we have proposed that the prereplicative cAMP surge (MacManus *et al.*, 1972) acts to inhibit the G1/S transition, and that the block in late G1 is overcome partly because of the increased R/C ratio occurring prereplicatively (Ekanger *et al.*, 1989). A similar mechanism may operate in the case of the isoproterenol-stimulated proliferation of parotid gland epithelium (Schwoch, 1987). In the case of human mammary carcinoma cell lines the findings are controversial (Handschin & Eppenberger, 1979; Eppenberger *et al.*, 1980; Prasad, 1981; Handschin *et al.*, 1983; Israeli *et al.*, 1985). Using an oestrogen responsive MCF-7 cell line, we first failed to obtain effects on DNA replication of cAMP elevating agents and cAMP analogues. However, when the phosphodiesterase activity of the cells was blocked a clear inhibition of DNA replication by cAMP could be demonstrated. Furthermore, microinjection of the C subunit of cAMP-dependent protein kinase led to inhibition of DNA replication (O. Vintermyr, A. Aakvaag & S.O. Døskeland, unpublished observations). The current evidence suggest to us that the growth associated increase of R/C ratio observed in liver (Ekanger *et al.*, 1989), parotid (Schwoch, 1987), and uterus and mammary carcinoma (the present study) serves to protect cells against negative modulation of DNA replication by cAMP. The importance of downregulation of cAK for cell cycle progression is further supported by a recent study using microinjection of an inhibitor of cAK into fibroblasts (Lamb *et al.*, 1991).

The mechanism of the increased R/C ratio may in principle be an increase of either RI or RII or a decrease of C subunit. In the case of the oestrogen-stimulated uterus and DMBA-induced mammary carcinomas the increase was mainly due to increased expression of RI (Figures 1,4). In mammary carcinomas from animals treated with cAMP-analogue RII and to a lesser extent RI showed increased expression (Table II). This difference suggests that the oestrogen-dependent overexpression of RI was not secondary to increased cAMP. In growth-stimulated DMBA-induced mammary tumours, both the RI and RII levels were higher than in oestrogen-deprived tumours (Table I), a situation reminiscent of the findings in the prereplicative regenerating rat liver, where both RI and RII rose to increase the R/C ratio (Ekanger *et al.*, 1989). It is noteworthy that a decrease of C did not contribute to the growth-associated increased R/C ratio in the DMBA-tumours (Table I). A slight decrease of C contributed to the increased R/C-ratio in prereplicative regenerating rat liver (Ekanger *et al.*, 1989), whereas decreased C was the main contributor to the increased R/C-ratio in isoproterenol-stimulated rat parotid cells (Schwoch, 1987), cAMP-stimulated hepatocytes in primary culture (Houge *et al.*, 1990*a*) and porcine kidney cells (Hemmings, 1986), e.g. in cells stimulated by cAMP. However, a decrease in C due to strong cAMP-stimulation is not compulsory. In this study, no decrease in C was found after treating uncastrated rats with cAMP-analogue (Table II). Similar findings have been done in various cell culture systems (Prasad, 1981; Lohmann & Walter, 1984; Gross *et al.*, 1990; Landmark *et al.*, 1991; Lanotte *et al.*, 1991).

A parameter which has been positively associated with growth in studies dating back more than a decade (Døskeland *et al.*, 1975; Russel, 1978; Cho-Chung, 1990) is the RI/RII ratio. In the present study the oestrogen-induced burst of DNA-synthesis was associated with increased RI/RII ratio (Figures 1,4), but the correlation was too weak to be statistically significant for the DMBA-tumours (Table I). The latter finding was somewhat suprising. In human mammary carcinomas the RI/RII ratio was found to be higher than in adenomas as judged by DEAE cellulose chromatography (Eppenberger *et al.*, 1980). It may be noted, as a general precaution when comparing studies of cAK isozyme expression, that the use of DEAE-cellulose to separate RI-holoenzyme from RII-holoenzyme is more error-prone (Malkinson *et al.*, 1983) than the use of antibodies (Ekanger & Døskeland, 1987). In non-steroid sensitive systems a positive correlation between RI and growth is supported by the following findings: cAMP analogue combinations preferentially activating type I isozyme of cAMP kinase were more efficient stimulators of thyrocyte DNA replication than type II directed combinations (Van Sande *et al.*, 1989); prolonged treatment with a thyroid stimulating hormone led to a parallel decrease of RI and the ability to respond to cAMP with increased proliferation (Breton *et al.*, 1989) and HL-60 leukaemia cells transfected with antisense oligodeoxynucleotide directed against RIα showed decreased growth (Tortora *et al.*, 1991). The uterine RI/RII ratio stayed elevated after the initial burst of oestrogen-induced DNA replication, i.e. when the uterus grew by hypertrophy rather than hyperplasia (Figure 1). Such a hypertrophy-associated increase of RI/RII ratio has also been found in the heart (Russel, 1978) and liver (Ekanger *et al.*, 1988). A possible explanation, supported by the findings in rat liver (Ekanger *et al.*, 1988), is that hypertrophying cells preferentially increase RI to keep their cytoplasmic concentration of R on a constant level. A preferential increase of RI was also found in transfected fibroblasts overexpressing the C subunit (Uhler & McKnight, 1987). It appears thus that although the present study contributes additional examples of association between increased RI/RII ratio and increased DNA synthesis and cell hypertrophy, such a correlation is not obligatory (e.g. Wittmaack, *et al.*, 1983).

This study did not provide indication of differential cAK subtype function in relation to growth, based on mRNA studies (Figure 3). If the mRNA level of Cα is used as a reference, the only clear evidence of differential mRNA expression found was a three fold increase in uterine RIIβ-mRNA 12 h after onset of high-dose oestrogen stimulation (Figure 3). High inducibility of RIIβ-mRNA has also been found in Friends erythroleukaemia cells, where RIIβ-mRNA was stabilised post-transcriptionally after activation of cAK (Gross *et al.*, 1990). It is not known if the increase in RIIβ-mRNA can be linked to increased cAMP level in our case. It is noteworthy that RIβ-mRNA was detected on conventional Northern blots in the uterus. RIβ-mRNA has previously only been found in the brain and germ cells (Clegg *et al.*, 1988; Massa *et al.*, 1990), and the significance of its expression in uterus is not known. However, recent experiments with holoenzymes reconstituted with RIα and RIβ, have shown the latter to be more sensitive to activation by cAMP (Cadd *et al.*, 1990). An overexpression of RIβ relative to RIα would therefore be expected to lower the threshold of the kinase towards activation by cAMP.

In the clinical setting the oestrogen receptor/R was a better parameter than oestrogen receptor/protein in predicting a tumour's response to endocrine therapy (Kvinnsland *et al.*, 1983). In addition the parameter R/protein (or total cAMP binding capacity) has been shown to be an independent prognostic factor for patients with early breast cancer (Miller *et al.*, 1990). In the present experimental study the ratios R/protein and R/C were tightly associated because the C subunit level was nearly constant (Figure 4; Table I). As reviewed above there is an increasing number of *in vivo* examples of increased R/C ratio in cells in transition from a resting state to a proliferating state (Figures 1,4; Table I). The increased R/C will protect the cells from negative regulation of proliferation by cAMP (Døskeland *et al.*, 1991). Possibly, mammary tumours with increased R expression have increased resistance to endocrine therapy.

The parameter R/protein depends on the determination of protein in an extract of tumour which may contain extracellular proteins and debris of protein nature from necrotic cells. This may explain why the R/protein values differ in different parts of the same tumour (Miller *et al.*, 1985). Since the amount of C subunit reflects the content of a cell protein, and the ratio between R and C subunits has biological significance, the R/C ratio may be a better parameter than R/protein for clinical purposes.

It was noted that ageing of tissue extracts led to rapid loss of immunoreactive RII, whereas total R (determined by direct cAMP binding capacity) was much more stable. This was presumably because tissue proteases clipped the cAMP binding domain from the aminoterminal domain containing the epitopes recognised by the antibodies. In the clinical setting, proteolysis can be a major problem for the accurate determination of RI and especially RII. In the present study several precautions were taken to avoid the loss of immunoreactive R: The tissue was freeze-clamped at the temperature of liquid nitrogen, grinded in liquid nitrogen and kept in that medium until homogenisation. Special care was taken to inhibit proteases during the homogenisation and immunoprecipitation, involving the use of an extensive protease-inhibitor cocktail (see methods) and doing all the work on ice or in the cold-room. As an extra precaution the rats in this study were intraperitoneally injected with leupeptin 1 h before tumour removal.

In summary, the direct determination of R and C is simpler and 'safer' than the separate measurement of RI and RII. RI, RII or RI/RII shows no better correlation with growth than R or R/C (Table I). The R level should be referred to the C level to calculate the biologically significant R/C ratio and to have a reference for R that is methodologically less problematic than protein measurements.

The technical assistance of Erna Finsås was highly appreciated. This work was supported by the Norwegian Cancer Society (DNK) and the F. Meltzer Foundation.

### References

BEEBE, J., ØYEN, O., SANDBERG, M., FRØYSA, A., HANSSON, V. & JAHNSEN, T. (1990). Molecular cloning of an unique, tissue-specific protein kinase (Cγ) from human testis–representing the third isoform for the catalytic subunit of cAMP-dependent protein kinase. *Mol. Endocrinol.*, **4**, 465–475.

BEEBE, S.J. & CORBIN, J.G. (1986). Cyclic nucleotide-dependent protein kinases. In *The Enzymes* vol. 17A, Boyer, P.D. & Krebs, E.G. (eds) pp. 43–111. Academic Press: Orlando/London.

BODWIN, J.S., CLAIR, T. & CHO-CHUNG, Y.-S. (1980). Relationship of hormone dependency to estrogen receptor and adenosine 3':5'-cyclic-monophosphate binding proteins in rat mammary tumors. *J. Natl Cancer Inst.*, **64**, 395–398.

BOSHART, M., WEIH, F., NICHOLS, M. & SCHÜTZ, G. (1991). The tissue-specific extinguisher locus TSE1 encodes a regulatory subunit of cAMP-dependent protein kinase. *Cell*, **66**, 849–859.

BOYNTON, A.L. & WHITFIELD, J.F. (1983). The role of cAMP in cell proliferation: A critical assessment of evidence. *Adv. Cyclic Nucleotide Res.*, **15**, 193–294.

BRADFORD, M. (1976). A rapid and sensitive method for the quantification of microgram quantities of protein utilizing the principle of protein-dye binding. *Anal. Biochem.*, **72**, 248–254.

BRETON, M.F., ROGER, P.P., OMRI, B., DUMONT, J.E. & PAVLOVIC-HOURNAC, M. (1989). Thyrotropin but not epidermal growth factor down-regulates the isozyme I (PKA I) of cyclic AMP-dependent protein kinase in dog thyroid cells in primary cultures. *Mol. Cell. Endocrinol.*, **61**, 49–55.

BRØNSTAD, G.O., SAND, T.-E. & CHRISTOFFERSEN, T. (1983). Bidirectional concentration-dependent effects of glucagon and dibutyryl cAMP on DNA synthesis in cultured rat hepatocytes. *Biochem. Biophys. Acta*, **763**, 58–63.

CADD, G.G., UHLER, M.D. & MCKNIGHT, G.S. (1990). Holoenzymes of cAMP-dependent protein kinase containing the neural form of TypeI regulatory subunit have an increased sensitivity to cyclic nucleotides. *J. Biol. Chem.*, **265**, 19502–19506.

CHO-CHUNG, Y.S. (1990). Role of cyclic AMP receptor proteins in growth, differentiation, and suppression of malignancy: new approaches to therapy. *Cancer Res.*, **50**, 7093–7100.

CHOMCZYNSKY, P. & SAACHI, N. (1987). Single-step method of RNA isolation by acid guanidinium thiocytanate-phenol-chloroform extraction. *Anal. Biochem.*, **162**, 156–159.

CLEGG, C.H., CADD, G. & MCKNIGHT, G.S. (1988). Genetic characterization of a brain-specific form of the type I regulatory subunit of cAMP-dependent protein kinase. *Proc. Natl Acad.Sci.*, **85**, 3703–3707.

COHEN, L.A. & CHAN, P.-C. (1975). Intracellular cAMP levels in normal rat mammary gland and adenocarcinoma *in vivo* vs *in vitro*. *Life Sci.*, **16**, 107–115.

DØSKELAND, S.O., KVINNSLAND, S. & UELAND, P.M. (1975). Protein kinases activated by cAMP in the genital tract of spayed mice treated with estradiol-17β. *J. Reprod. Fertil.*, **44**, 207–216.

DØSKELAND, S.O., BØE, R., BRULAND, T., VINTERMYR, O.K., JASTORFF, B. & LANOTTE, M. (1991). Criteria used to judge that a cellular response is mediated by cAMP. In *Methodological Surveys in Biochemistry and Analysis* vol. 21, Reid, E. (ed) pp. 103–114. The Royal Society of Chemistry, UK.

EDELMAN, A., BLUMENTHAL, D.K. & KREBS, E.G. (1987). Protein serine/threonine kinases. *Ann. Rev. Biochem.*, **56**, 567–613.

EKANGER, R. & DØSKELAND, S.O. (1987). Use of antibody-sepharose columns to study hormonal activation of cAMP-dependent protein kinase isozymes. *Methods Enzymol.*, **159**, 97–104.

EKANGER, R., VINTERMYR, O.K. & DØSKELAND, S.O. (1988). The amounts of rat liver cAMP-dependent protein kinase I and II are differentially regulated by diet. *Biochem. J.*, **256**, 447–452.

EKANGER, R., VINTERMYR, O.K., HOUGE, G., SAND, T.-E., SCOTT, J.D., KREBS, E.G., EIKHOM, T., CHRISTOFFERSEN, T., ØGREID, D. & DØSKELAND, S.O. (1989). The expression of cAMP-dependent protein kinase subunits is differentially regulated during liver regeneration. *J. Biol. Chem.*, **264**, 4374–4382.

EPPENBERGER, U., BIEDERMANN, K., HANDSCHIN, J.C., FABBRO, D., KÜNG, W., HUBER, P.R. & ROOS, W. (1980). Cyclic-AMP-dependent protein kinase type I and type II and cyclic AMP binding in human mammary tumors. *Adv. Cyclic Nucleotide Res.*, **12**, 123–128.

ESCRICH, E. (1987). Validity of the DMBA-induced mammary cancer model for the study of human breast cancer. *Int. J. Biol. Markers*, **2**, 197–206.

GOTTESMAN, M.M. & FLEISCHMANN, R.D. (1986). The role of cAMP in regulating tumor cell growth. *Cancer Surv.*, **5**, 291–308.

GROSS, R.E., LU, X.Y. & RUBIN, C.S. (1990). Regulation of the expression of the regulatory subunit of cAMP-dependent protein kinase-II-beta in friend erythroleukemic cells – evidence for post-transcriptional control and a central role for the C-subunit. *J. Biol. Chem.*, **265**, 8152–8158.

HANDSCHIN, J.C. & EPPENBERGER, U. (1979). Altered cellular ratio of type I and type II cyclic AMP-dependent protein kinase in human mammary tumors. *FEBS Lett.*, **106**, 301–304.

HANDSCHIN, J.C., HANDLOSER, K., TAKAHASHI, K. & EPPENBERGER, U. (1983). Cyclic adenosine 3':5'-monophosphate receptor proteins in dysplastic and neoplastic human breast tissue cytosol and their inverse relationship with estrogen receptors. *Cancer Res.*, **43**, 2947–2954.

HEMMINGS, B.A. (1986). cAMP mediated proteolysis of the catalytic subunit of cAMP-mediated protein kinase. *FEBS Lett.*, **196**, 126–130.

HOFMANN, F., BEAVO, J.A., BECHTEL, P.J. & KREBS, E.G. (1977). Concentrations of cAMP-dependent protein kinase subunits in various tissues. *J. Biol. Chem.*, **252**, 1441–1447.

HOUGE, G., VINTERMYR, O.K. & DØSKELAND, S.O. (1990*a*). The expression of cAMP-dependent protein kinase in primary rat hepatocyte cultures. Cyclic AMP down-regulates its own effector system by decreasing the amount of catalytic subunit and increasing the inhibitory (R) subunits of cAMP-dependent protein kinase. *Mol. Endocrinol.*, **4**, 481–488.

HOUGE, G., STEINBERG, R.A., ØGREID, D. & DØSKELAND, S.O. (1990*b*). The rate of recombination of the subunits (RI and C) of cAMP-dependent protein kinase depends on whether one or two cAMP molecules are bound per RI monomer. *J. Biol. Chem.*, **265**, 19507–19516.

HUGGINS, C., GRAND, L.C. & BRILLANTE, F.P. (1961). Mammary cancer induced by single feeding of polynuclear hydrocarbons and its suppression. *Nature*, **189**, 204.

ISRAELI, E., RAZ, B., KERNER, H. & BARZILAI, D. (1985). Cyclic nucleotide levels in human breast cancer and in rat mammary tissues during tumor development. *Breast Cancer Res. Treat.*, **6**, 241–248.

JONES, K.W., SHAPERO, M.H., CHEVRETTE, M. & FOURNIER, R.E.K. (1991). Subtractive hybridization cloning of a tissue-specific extinguisher: TSE1 encodes a regulatory subunit of protein kinase A. *Cell*, **66**, 861–872.

KVINNSLAND, S., EKANGER, R., DØSKELAND, S.O. & THORSEN, T. (1983). Relationship of cyclic AMP binding capacity and oestrogen receptor to hormone sensitivity in human breast cancer. *Breast Cancer Res. Treat.*, **3**, 67–72.

LAMB, N.J.C., CAVADORE, J.-C., LABBE, J.-C., MAURER, R.A. & FERNANDEZ, A. (1991). Inhibition of cAMP-dependent protein kinase plays a key role in the induction of mitosis and nuclear envelope breakdown in mammalian cells. *EMBO J.*, **10**, 1523–1553.

LANDMARK, B.F., FAUSKE, B., ESKILD, W., SKÅLHEGG, B., LOHMANN, S., HANSSON, V., JAHNSEN, T. & BEEBE, S.J. (1991). Identification characterization and hormonal regulation of 3′,5′-cyclic adenosine monophosphate-dependent protein kinase in rat sertoli cells. *Endocrinology*, **129**, 2345–2354.

LANOTTE, M., HERMOUET, S., HOUGE, G., VINTERMYR, O.K. & DØSKELAND, S.O. (1991). The selective activation of cAMP-dependent protein kinase by site specific analog pairs induces a cytolytic process in a myeloid leukemic cell line. *J. Cell. Physiol.*, **146**, 73–80.

LAVIA, L.A., SHIDELER, C., FARLEY, N., WALKER, N., FIELDS, W. & ROBERTS, D.K. (1984). Uterine growth responses of the mature castrate rat to estradiol-17$\beta$. *Steroids*, **43**, 663–675.

LEE, A.E. (1972). Cell division and DNA synthesis in the mouse uterus during continuous oestrogen treatment. *J. Endocrinol.*, **55**, 507–513.

LOHMANN, S.M. & WALTER, U. (1984). Regulation of cellular and subcellular concentrations and distribution of cyclic nucleotide-dependent protein kinases. *Adv. Cyclic Nucleotide Res.*, **18**, 63–117.

MACMANUS, J.P., BRACELAND, B.M., YOUNDALE, T. & WHITEFIELD, J.F. (1972). Increase in rat liver cyclic AMP concentrations prior to the initiation of DNA synthesis following partial hepatectomy or hormone infusion. *Biochem. Biophys. Res. Commun.*, **49**, 1201–1207.

MALKINSON, A.M., BEER, D.S., WEHNER, J.M. & SHEPPARD, J.R. (1983). Elution of the regulatory subunit of cAMP-dependent protein kinase type I derived from epididymal fat within the type II isozyme chromatographic peak. *Biochem. Biophys. Res. Commun.*, **112**, 214–220.

MARTIN, L., FINN, C.A. & TRINDER, G. (1973). Hypertrophy and hyperplasia in the mouse uterus after estrogen treatment: an autoradiographic study. *J. Endocrinol.*, **56**, 133–144.

MASSA, J.S., FELLOWS, R.E. & MAURER, R. (1990). Rat RI$\beta$ isoform of type I regulatory subunit of cyclic adenosine monophosphate-dependent protein kinase: cDNA sequence analysis, mRNA tissue specificity, and rat/mouse difference in expression in testis. *Mol. Reprod. Develop.*, **26**, 129–133.

MEITES, J. (1972). The relation of estrogen and prolactin to mammary tumorigenesis in the rat. In *Estrogen Target Tissues and Neoplasia*, Dao, T.L., (ed.) pp. 275–286. The University of Chicago Press: Chicago.

MILLER, W.R., SENBANJO, R.O., TELFORD, J. & WATSON, D.M.A. (1985). Cyclic AMP binding proteins in human breast cancer. *Br. J. Cancer*, **52**, 531–535.

MILLER, W.R., ELTON, R.A., DIXON, J.M., CHETTY, U. & WATSON, D.M.A. (1990). Cyclic AMP binding proteins and prognosis in breast cancer. *Br. J. Cancer*, **61**, 263–266.

PATTERSON, M.K. (1979). Measurement of growth and viability of cells in culture. *Methods Enzymol.*, **58**, 141–152.

PRASAD, K.N. (1981). Involvement of cyclic nucleotides in transformation. In *The Transformed Cell*, pp. 235–266. Academic Press: Orlando/London.

PRASHAD, N. (1981). Induction of free cAMP-binding protein by dibutyryl cAMP in neuroblastoma cells. *Cold Spring Harbor Symposia on Cell Proliferation*, **83**, 159–178.

RUSSEL, D.H. (1978). Type I cyclic AMP-dependent protein kinase as positive effector of growth. *Adv. Cyclic Nucleotides Res.*, **9**, 493–506.

SCHWOCH, G. (1987). Selective regulation of the amount of catalytic subunit of cAMP-dependent protein kinases during isoprenaline-induced growth of the rat parotid gland. *Biochem. J.*, **248**, 243–250.

STROMSHAK, F., LEAKE, R., WERTZ, N. & GORSKI, J. (1976). Stimulatory and inhibitory effects of estrogen on uterine DNA synthesis. *Endocrinology*, **18**, 1501–1511.

THORESEN, H., SAND, T.-E., REFSNES, M., DAJANI, O.F., GUREN, T.K., GLADHAUG, I.P., KILLI, A. & CHRISTOFFERSEN, T. (1990). Dual effects of glucagon and cyclic AMP on DNA synthesis in cultured rat hepatocytes: stimulatory regulation in early G1 and inhibition shortly before the S phase entry. *J. Cell. Physiol.*, **144**, 523–530.

TORTORA, G., YOKOZAKI, H., PEPE, S., CLAIR, T. & CHO-CHUNG, Y.S. (1991). Differentiation of HL-60 leukemia by type I regulatory subunit antisense oligodeoxynucleotide of cAMP-dependent protein kinase. *Proc. Natl Acad. Sci. USA*, **88**, 2011–2015.

UHLER, M.D. & MCKNIGHT, G.S. (1987). Expression of cDNAs for two isoforms of the catalytic subunit of cAMP-dependent protein kinase. *J. Biol. Chem.*, **262**, 15202–15207.

VAN SANDE, J., LEFORT., A., BEEBE, S., ROGER, P., CORBIN, J. & DUMONT, J.E. (1989). Pairs of cyclic AMP analogs, that are specifically synergistic for type I and type II cAMP-dependent protein kinases, mimic thyrotropin effects on the function, differentiation expression and mitogenesis of dog thyroid cells. *Eur. J. Biochem.*, **183**, 699–708.

VINTERMYR, O.K. & DØSKELAND, S.O. (1987). Cell cycle parameters of adult rat hepatocytes in a defined medium. A note on the timing of nucleolar DNA replication. *J. Cell. Physiol.*, **132**, 12–21.

VINTERMYR, O.K., MELLGREN, G., BØE, R. & DØSKELAND, S.O. (1989). Cyclic AMP acts synergistically with dexamethasone to inhibit the entrance of cultured rat hepatocytes into S phase. *J. Cell. Physiol.*, **141**, 371–382.

WATSON, D.M.A., HAWKINS, R.A., BUNDRED, N.J., STEWART, H.J. & MILLER, S.R. (1987). Tumour cyclic AMP binding proteins and endocrine responsiveness in patients with inoperable breast cancer. *Br. J. Cancer*, **56**, 141–142.

WITTMAACK, F.M., WEBER, W. & HILZ, H. (1983). Isoenzymes of cAMP-dependent protein kinase in developing rat liver and in malignant hepatic tissues. *Eur. J. Biochem.*, **129**, 669–674.